# Assessing Behavioural Manifestations Prior to Clinical Diagnosis of Huntington Disease: "Anger and Irritability" and "Obsessions and Compulsions"

**DOI:** 10.1371/currents.RRN1241

**Published:** 2011-07-11

**Authors:** Anthony L Vaccarino, Anonymous, Karen E. Anderson, Beth Borowsky, Emil Coccaro, David Craufurd, Jean Endicott, Joseph Giuliano, Mark Groves, Mark Guttman, Aileen K Ho, Peter Kupchak, Jane S. Paulsen, Matthew S. Stanford, Daniel P van Kammen, David Watson, Kevin D Wu, Ken Evans

**Affiliations:** ^*^Research Methods, Ontario Cancer Biomarker Network, Toronto, Ontario, Canada; ^†^Department of Psychiatry and Department of Neurology, University of Maryland, School of Medicine, Baltimore, MD USA; ^‡^Translational Medicine, CHDI Foundation, Inc., Princeton NJ; ^§^Research Psychiatrist; ^¶^University of Manchester, Manchester Academic Health Sciences Centre and Central Manchester University Hospitals NHS Foundation Trust, Manchester, UK; ^#^Department of Psychiatry, Columbia University College of Physicians and Surgeons, New York, New York; ^**^CHDI Foundation, Inc.; ^††^Departments of Neurology and Psychiatry, Beth Israel Medical Center, New York, NY; ^‡‡^Division of Neurology, Department of Medicine, University of Toronto, Toronto, Ontario Canada; ^§§^School of Psychology and Clinical Language Sciences, University of Reading, U.K.; ^##^Department of Psychiatry, The University of Iowa Carver College of Medicine, Iowa City, IA, USA; ^***^Department of Psychology & Neuroscience, Baylor University, Waco, TX; ^†††^CNS Drug Development consultant; ^‡‡‡^Department of Psychology, University of Notre Dame, Notre Dame, Indiana, USA and ^§§§^Department of Psychology, Northern Illinois Unversity, DeKalb IL USA

## Abstract

The Functional Rating Scale Taskforce for pre-Huntington Disease (FuRST-pHD) is a multinational, multidisciplinary initiative with the goal of developing a data-driven, comprehensive, psychometrically sound, rating scale for assessing symptoms and functional ability in prodromal and early Huntington disease (HD) gene expansion carriers. The process involves input from numerous sources to identify relevant symptom domains, including HD individuals, caregivers, and experts from a variety of fields, as well as knowledge gained from the analysis of data from ongoing large-scale studies in HD using existing clinical scales. This is an iterative process in which an ongoing series of field tests in prodromal (prHD) and early HD individuals provides the team with data on which to make decisions regarding which questions should undergo further development or testing and which should be excluded. We report here the development and assessment of the first iteration of interview questions aimed to assess "Anger and Irritability" and "Obsessions and Compulsions" in prHD individuals.

## Introduction

Earliest clinical manifestations of Huntington disease (HD) are poorly characterized, and there is a need for clinical scales specifically designed to measure early changes in HD gene expansion carriers. The Functional Rating Scale Taskforce for pre-Huntington Disease (FuRST-pHD) is a multinational, multidisciplinary collaboration to develop a valid functional rating scale to assess severity of manifestations in HD gene expansion carriers who do not yet meet criteria for a formal clinical diagnosis (prodromal HD or prHD) or are early manifest.[1] Such a measurement tool is essential to better understand the earliest manifestations of HD and to evaluate novel therapies early in the course of disease. 

FuRST-pHD has established an inclusive process using input from numerous sources, including prHD and early HD individuals, caregivers, and experts from a variety of fields, as well as from ongoing large-scale HD studies using existing clinical scales.[1] As part of the process, an inclusive series of “Working Groups” of individuals with clinical and/or scale development expertise have been established to review existing data and develop interview questions within the specific domain under study. Once these interview questions are developed, they are distributed to trained raters for beta testing in gene expansion carriers. This is an iterative process, in which changes or deletions (as appropriate) are made based on empirical evidence obtained during field testing; the modified questions are then tested during subsequent iterations so that the list can ultimately be winnowed to select optimal items for scale inclusion. 

"Anger and Irritability" and "Obsessions and Compulsions" were identified as potential early symptoms exhibited by gene carriers for which proven measures are unavailable for this population. We report here the development and assessment of the first iteration of interview questions aimed to assess "Anger and Irritability" and "Obsessions and Compulsions" in prHD.

## Methods

Two-day Working Group meetings of individuals with a broad range of relevant expertise were held in Toronto, Canada to assess Anger and Irritability (October 22–23, 2008) and Obsessions and Compulsions (October 24–25, 2008). The working groups' charges were to review available evidence and provide input into development of interview questions to assess "Anger and Irritability" and "Obsessions and Compulsions" in prHD.

### Evidence Reviewed

#### Data Mining.

Although existing tools were not specifically designed to assess early manifestations in HD gene carriers, studies using such measures can nevertheless provide rich and useful information about the expression of these changes in the target population, the differentiation of early manifestations from those expressed in advanced disease, or similar symptoms seen in other disorders. Fortunately, there are a number of ongoing studies investigating the symptomatology and progression of prHD and HD that are accessible to the FuRST pHD program, including PREDICT-HD, REGISTRY, and TRACK-HD. These data were reviewed and considered by the working group in developing the interview questions.

#### Patient and Companion Input.

The FDA views input from participants, caregivers and family members as an essential element in developing valid clinical assessment tools.[2] To ensure that the scale reflects concepts that are important from the participant's perspective, patient/companion focus groups were held to identify early symptoms experienced by HD gene carriers. The focus groups were held in a number of countries using the local languages (France, Netherlands, United Kingdom, United States, Portugal, and Spain) with all participants (prHD, early HD and companions) being asked a series of open-ended questions related to symptom occurrence in prHD. All focus group sites had IRB/EC approval, and all participants provided informed consent. These data were used to advance saturation of symptom assessment and were considered in development of the interview questions (Figure 1).

**Figure:**
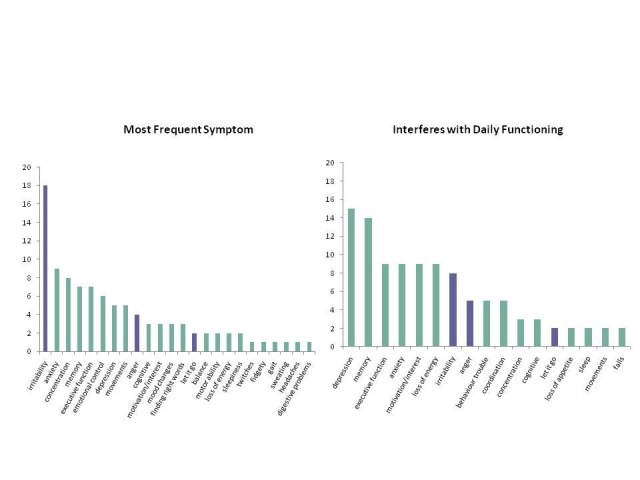
**Figure 1**. Client/caregiver focus groups: Symptom frequency and impact (French, Dutch, USA, Spanish, Portuguese; number reporting)

#### Expert Opinion and Experience of Participants.

In addition to reviewing existing data, working group participant experiences and opinion were also discussed.

### Development of Interview Questions 

Based on data mining and input from gene expansion carriers, caregivers, experts, and literature, symptom domains and definitions are identified that are thought to be important to prHD. These diverse sources of information provided an excellent starting point for establishing which symptoms are important to participants. After review of existing data, relevant symptom domains were identified and interview questions were developed to assess specific symptoms within each cluster (and determine their severity) in prHD (Figure 2). 

**Figure:**
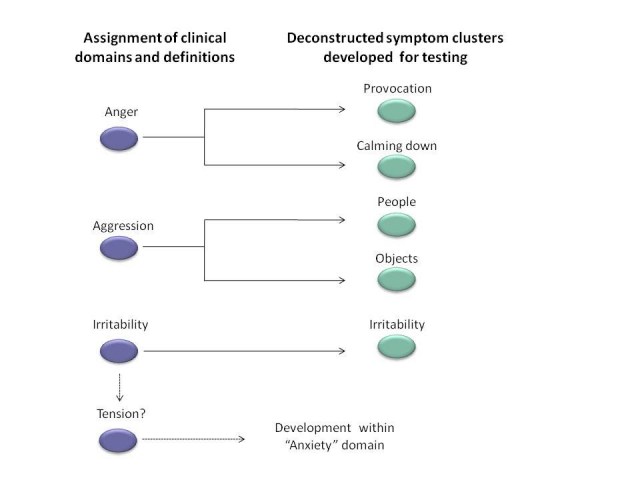
**Figure 2**. Development of interview questions to assess anger and irritability. Based on patient, caregiver, expert, and literature input, symptom domains are identified that are thought to be important to prHD. The Working Groups identify the constituent symptoms based on the words used by patients and caregivers, conventional definitions and their clinical and scientific opinion. Interview questions are then developed for each of these narrowly defined symptoms.

The FuRST-pHD has adopted a semi-structured clinician-administered interview similar to that used for the GRID-HAMD. The GRID format directs the rater to score symptom frequency and intensity separately, while giving them clear scoring anchors, a semi-structured interview guide, and overall definitions. This method has been employed successfully and is user-friendly, with acceptable agreement among independent raters.[3] The working group developed interview questions, including structured interview guides, scoring conventions, scoring anchors, and symptom definitions. Following the meeting, draft interview questions were circulated for comment on a shared internet site (Sharepoint).

Based on a review of the evidence, 7 interview questions assessing "Anger and Irritability" and 5 interview questions assessing "Obsessions and Compulsions" were developed for field testing (Table 1).

**Table 1**. Interview Questions

**Table:** 

**Interview Question**	**Description/definition**
**Anger/irritability**	Assesses both irritability (proneness to annoyance) as well as anger (strong displeasure with self or others, accompanied by signs of autonomic arousal).
**Ease of Anger - provocation**	Assesses the ease with which individual becomes annoyed or angered when they are confronted or provoked by others.
**Ease of anger - stressor**	Assesses the ease with which individual becomes annoyed or angered when under conditions of stress or confronted with day-to-day hassles.
**Ease of calming down**	Assesses the ease with which individual can calm down after they have become angered or annoyed.
**Calm down time**	Assesses the amount of time it takes individual to calm down once annoyed or angered.
**Aggression - People**	Assesses the individual’s aggressive behaviors toward other people.
**Aggression - Animals/Objects**	Assesses the individual’s aggressive behaviors toward animals or objects.
**Repetitive Thoughts**	Assesses the experience of repetitive thoughts.
**Compromise**	Assesses ability to compromise.
**Perseveration - Tasks**	Assesses fixation on tasks and the difficulty in shifting away from those tasks.
**Perseveration - Topics of conversation**	Assesses fixation on specific topics of conversation and the difficulty in shifting away from those conversations.
**Mental inflexibility/Rigidity**	Assesses mental rigidity and inflexibility; defined as insisting on having own way, or have increasing difficulty adapting to new or changing circumstances.

### Field Testing of Interview Questions

Field testing of interview questions in prHD (UHDRS Diagnostic Confidence Level < 4) and early HD (within 5 years from onset of clinical motor signs) was conducted within the PREDICT-HD program and at independently contracted sites. All data collection sites had IRB/EC approval, and all participants provided informed consent. Prior to conducting the clinical interview, all raters were trained (via webinar or in person) to ensure that all trainees had an adequate conceptual understanding for administering and scoring each of the items. A minimum sample size of 100 was targeted.

### Data Analysis 

The distribution of the composite score for each individual item was compiled, and summary statistics associated with each item score were computed.  Distributions of item scores for prHD and HD subgroups were statistically compared using the non-parametric Mann-Whitney U test.  

Non-parametric item response analyses were performed to determine the relationship between scores on the individual interview questions and total score. Item Response Theory (IRT) has been demonstrated to be useful in evaluating the performance of individual items (symptoms) on rating scales, by assessing the relationship between a score assigned to an item and the overall severity of the disease.[4] [5] IRT software (TESTGRAF) was used to generate Option Characteristic Curves (OCCs) that display the probability of a particular option score (i.e., a score of 0, 1, 2, 3, 4) on each Interview Question as a function of overall level of severity. In the present analyses, total score of all interview questions was used as a measure of severity. To illustrate this, Figure 3 depicts a hypothetically ‘‘ideal’’ item from an item response perspective, which is characterized by a clear identification of the range of severity scores over which an option is most likely to be endorsed, rapid changes in the curves that correspond to changes in severity, and an orderly relationship between the weight assigned to the option and the region of severity over which an item is likely to be endorsed. As such, OCCs provide a graphical representation of how informative a particular item (or symptom) is as a measure of illness. Frequency distribution of option scoring within each interview question were also generated.

**Figure:**
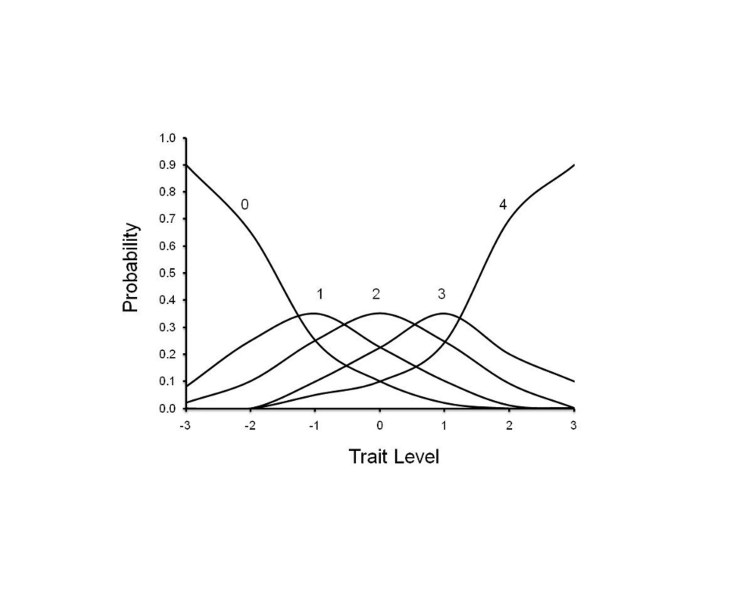
**Figure 3**. OCC for a hypothetically ‘‘ideal’’ item. The OCC shows the probability (*y*-axis) of endorsing a particular option for the item at different levels of the trait (*x*-axis).

Interview questions which were found to produce scoring and discrimination across ranges of overall severity were putatively selected for further testing. Scores for prHD and HD subjects were computed and compared statistically using the Mann-Whitney U test. The measure of internal consistency of the subscale was estimated using Cronbach's alpha, and item-total correlations between subscale scores and scores of individual questions not included in the subscale were computed.

## Results

A total of 225 CRFs were completed. The participant demographic characteristics are shown in Table 2.

**Table 2.** Demographic Characteristics 

**Table:** 

** **	**Total**	** prHD**	**HD**
**Sample size**	N=225	N=190 (84%)	N=35 (16%)
**Male gender**	N=98 (44%)	N=80 (42%)	N=18 (51%)
**Age**	44.4 (18-81)	42.2 (18-77)	49.9 (25-81)

A follow-up meeting (via webinar) was held with the working group to review data and make recommendations in moving forward, including item deletion and modification/refinement. The FDA PRO Guidance was used to guide the decision making process.[2]

OCCs and scoring frequency distributions were generated for each of the interview questions. Of the 12 tested, 6 interview questions were found to produce scoring and discrimination across ranges of overall severity:

### ✓  Anger/irritability

### ✓  Ease of Anger - provocation by other people

### ✓  Ease of Anger - stressor

### ✓  Ease of calming down 

### ✓  Calm down time

### ✓  Compromise

The internal consistency of these six items was high, as were the corrected item-total correlations (Table 3, shaded rows). Cronbach's alpha was 0.86 with respect to the entire study population, 0.85 with respect to the prHD subgroup, and 0.92 with respect to the HD subgroup. All corrected item-total correlations were 0.52 or higher with respect to the prHD subgroup and 0.67 or higher with respect to the HD subgroup (Table 3).

The mean total composite score with respect to the above 6 questions was 3.69 in prHD subjects and 3.70 in HD subjects; the difference in mean scores was not statistically significant (p = 0.81, Mann-Whitney U test). No significant differences were noted in scoring between prHD and HD for any of the 6 individual interview questions, with respect to either intensity or frequency of symptoms.

**Table 3.  **Correlations Between Interview Questions Scores

**Table:** 

Interview Question	Item-total correlation (all subjects)	Item-total correlation (prHD subjects)	Item-total correlation (HD subjects)
Anger/Irritability	0.71	0.70	0.79
Ease of anger - provocation	0.63	0.60	0.78
Ease of anger - stressor	0.70	0.68	0.75
Ease of calming down	0.69	0.64	0.92
Calm down time	0.69	0.68	0.78
Aggression - people	0.44	0.39	0.62
Aggression - animals/objects	0.36	0.38	0.29
Repetitive thoughts	0.29	0.34	0.14
Compromise	0.54	0.52	0.67
Perseveration - tasks	0.23	0.22	0.28
Perseveration - conversation	0.16	0.14	0.24
Mental inflexibility/rigidity	0.16	0.16	0.16

It was agreed that these 6 interview questions would be modified accordingly for testing in subsequent iterations; examination of the OCCs provided data on which decisions could be made as to where modifications should be made to improve item performance, including changes in wording and scoring options. For example, Figure 4 shows the OCCs for the Anger/Irritability question. The options with the highest probably of being scored for symptom intensity increased from "0" to "1" (mild: somewhat irritable, with minor autonomic arousal) to "2" (moderate: very irritable, definite autonomic arousal, but no overt aggression expressed); however, scores of "3" (severe: definite expression of anger, transient loss of control) and "4" (very severe: definite loss of control, throwing objects, injury to persons, animals, objects, or self) were rarely endorsed, suggesting that aggressive or violent outbursts are not typically characteristic symptoms in this population. Indeed, interview questions aimed to directly assess aggression (see Table 1) were hardly endorsed. Also of note was that the distribution of options scoring was similar in prHD and HD participants (Figure 4, as example), suggesting that these behavioral symptoms do not worsen in early HD or track with the development of early motor manifestations.[6] Finally, with respect to the frequency of symptoms, most symptoms (when present) were described as occurring occasionally (i.e., score of 1, see Figure 4), suggesting that the measurement of severity of anger/irritability may sufficiently be captured by symptom intensity alone. 

**Figure:**
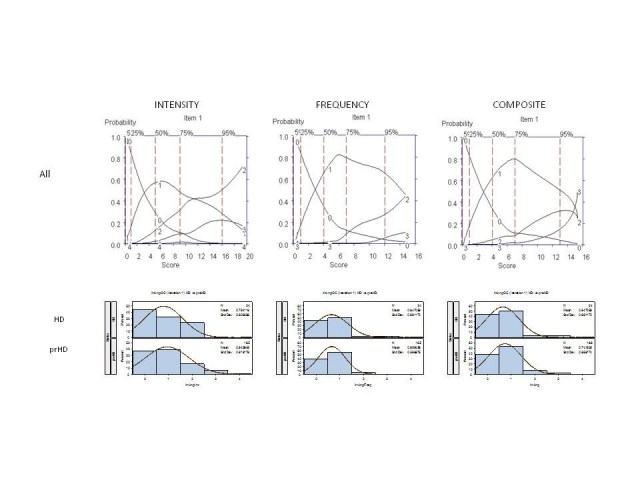
**Figure 4.** OCCs (All participants) and Frequency distribution (HD and prHD) for interview question assessing "anger and irritability." Data are presented separately for symptom intensity (left row), frequency (middle row), and composite (right row) scores.

The remaining 6 questions were very rarely endorsed and a score of zero had the highest probability of being scored across the full range of severity (Figure 5, as example): 

### ✗  Aggression - People

### ✗  Aggression - Animals/Objects

### ✗  Repetitive Thoughts

### ✗  Perseveration - Tasks

### ✗  Perseveration - Topics of conversation

### ✗  Mental inflexibility/Rigidity

The mean composite scores for these six questions were lower than those for the six highly-endorsed interview questions, with respect to the overall study population and the subgroup of prHD subjects (*p* < 0.05). The low frequency of response and poor discriminative properties limit the usefulness of these interview questions for assessment in prHD and early HD, including the measures of obsessive-compulsive behaviors. The only questions which discriminated between prHD and early HD subjects was "Perseveration - topics of conversation," with significantly higher scores in HD subjects with respect to both frequency (*p* < 0.05) and intensity (*p* < 0.05) of symptoms; however, this question was nonetheless very rarely endorsed in the subject population. For the other five items, there were no statistically significant differences in either the severity or frequency of symptoms between prHD and HD subjects (Figure 5, as example). In general, the correlations between each of these 6 individual composite scores and the total composite score from the 6 highly-endorsed interview questions were low (Table 3); the item-total correlation was high for "Aggression towards people" in the subset of prHD subjects, but this item was nonetheless rarely endorsed in the subject population.

**Figure:**
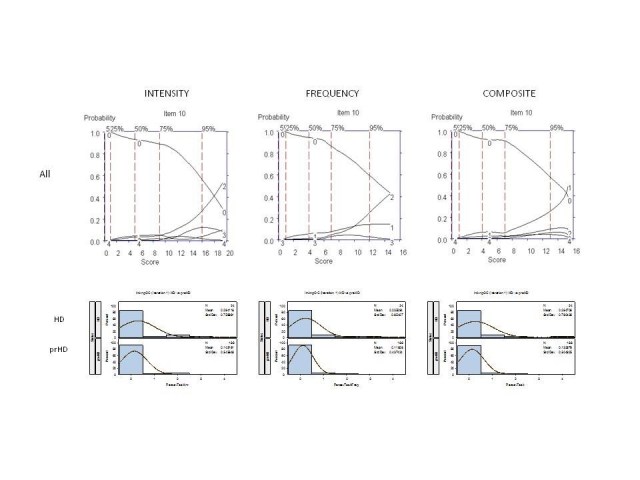
**Figure 5.** OCCs (All participants) and frequency distribution (HD and prHD) of representative rarely-endorsed interview question (perseveration-tasks). Data presented separately for symptom intensity (left row), frequency (middle row), and composite (right row) scores.

It was agreed that that these 6 questions should be removed from subsequent iterations on the basis of Relevance (Reported as not relevant by a large segment of the population of interest) and Response Range (A high percentage of patients respond at the floor) as outlined in Table 1 of the FDA PRO Guidance.[2]

## Discussion

FuRST-pHD has used an inclusive, iterative process to generate interview questions to assess symptoms in prodromal and early HD gene expansion carriers. While testing is still ongoing, it is clear that many CAG expanded individuals exhibit a range of behavioral manifestations prior to clinical diagnosis, but some symptoms are likely to be better candidates for inclusion in a final instrument than others. We report here the development and beta testing of first iteration interview questions designed to assess anger/irritability and obsessions/compulsions. Six questions have been selected for further testing, have been modified accordingly by the working groups, and are currently undergoing a second iteration of field testing. The results of the second iteration will be reported once completed. 

## Acknowledgments

CHDI Foundation, Inc. – a not-for-profit research organisation whose mission is to rapidly and collaboratively discover and develop therapies that slow the progression of Huntington’s disease – initiated and sponsored the development of the FuRST-pHD. We thank Jamie Levey for her help coordinating the European focus groups,  LaVonne Goodman for her help coordinating the USA focus groups, and Stacie Vik and Barbara McQuaid for administrative assistance.

## Funding Information 

FuRST-pHD is funded by CHDI. PREDICT-HD is supported by the National Institutes for Health, National Institute of Neurological Disorders and Stroke (NS40068) and CHDI Foundation, Inc.

## Competing Interests

The authors have declared that no competing interests exist.

## FuRST-pHD Core Team 

K Anderson, B Borowsky, K Evans, J Giuliano, M. Guttman. A Ho, JS Paulsen, T Sills, A Vaccarino, D van Kammen  

## Anger and Irritability Working Group

FuRST-pHD Core Team, E Coccaro, D Craufurd, J Endicott, M Groves, M Stanford

## Obsessions and Compulsions Working Group

FuRST-pHD Core Team, D Craufurd, J Endicott,  D Watson, K Wu,

## Statistics

S Gilbert-Evans, P Kupchak, T Sills, A Vaccarino,

## Contributing Field Testing Sites

PREDICT-HD: University of Iowa, Iowa City, Iowa, USA (Leigh J. Beglinger, PhD, Stephen Cross, BA, Mycah Kimble, BA, Pat Ryan, MSW, MA, Stacie M. Vik, BA, Thomas Wassink, MD); University of British Columbia, Vancouver, British Columbia, Canada (Lynn Raymond, MD, PhD, Rachelle Dar Santos, BSc and Joji Decolongon, MSC); Johns Hopkins University, Baltimore, Maryland, USA (Adam Rosenblatt, MD, Christopher A. Ross, MD, PhD, and Claire Welsh); Cambridge Centre for Brain Repair, Cambridge, UK (Roger A. Barker, BA, MBBS, MRCP, Sarah Mason, BSC, Anna Goodman, PhD, Rachel Swain and Anna DiPietro); Westmead Hospital, Sydney, Australia (Elizabeth McCusker, MD, Jane Griffith, RN, and David Gunn); Indiana University School of Medicine, Indianapolis, IN (Kimberly Quaid, PhD and Melissa Wesson, MS); Center for Movement Disorders, Markham, Ontario, Canada (Mark Guttman, MD, Irita Karmalkar, BA, Alanna Sheinberg, BA, and Adam Singer, BA); University of California, Los Angeles Medical Center, Los Angeles, California, USA (Susan Perlman, MD and Arik Johnson, PsyD); University of California San Francisco, California, USA (Michael D. Geschwind, MD, PhD and Jon Gooblar, BA); University of Rochester, Rochester, New York, USA (Amy Chesire, LCSW-R, MSG and Frederick Marshall, MD); Neurosciences Unit, Graylands, Selby-Lemnos & Special Care Health Services, Perth, Australia (Peter Panegyres, MB, BS, PhD, Elizabeth Vuletich, BSC, Steve Andrew); Washington University, St. Louis, Missouri, USA (Joel Perlmutter, MD and Stacey Barton, MSW, LCSW); Columbia University Medical Center, New York, New York, USA (Pietro Mazzoni, MD, PhD and Paula Wasserman, MA); Colorado Neurological Institute, Englewood, Colorado, USA (Diane Erickson, RN and Rajeev Kumar, MD); University of California Davis, Sacramento, California, USA (Vicki Wheelock, MD, Terry Tempkin, RNC, MSN); Baylor College of Medicine, Houston, Texas, USA (Joseph Jankovic, MD, Christine Hunter, RN, CCRC, and William Ondo, MD); Cleveland Clinic Foundation, Cleveland, Ohio, USA (Anwar Ahmed, PhD, Christine Reece, BS, Alexandra Bea, BA, Alex Bura, BA and Emily Newman, BA). OCBN Contracted: Birmingham and Solihull Mental Health, Birmingham, UK (Hugh Rickards, MD, Jenny Crooks, BA, Jan Wright, BA); Center for Movement Disorders, Markham, Ontario, Canada (Mark Guttman, MD, Irita Karmalkar, BA, and Alanna Sheinberg, BA, and Adam Singer, BA); University of Melbourne, AU (David Ames, MD, Edmond Chiu, MD, Phyllis Chua, MD, Olga Yastrubetskaya, PhD, Joy Preston, Anita Goh, D.Psych, and Angela Komiti, BS, MA); University of Iowa, Iowa City, IA, USA (Leigh Beglinger, PhD., Kevin Duff, PhD, Tom Wassink, MD, Patricia Ryan, MSW, MA, Stephen Cross, BA, Mycah Kimble, BA, Stacie Vik, BA); Huntington Disease Drug Works, Seattle, WA, USA (LaVonne Goodman, MD); North York General Hospital, Toronto. Ontario, Canada (Clare Gibbons, MS, Jeanne Kennedy, BScNEd, RN, and Wendy Meschino, MD).

## Focus Groups

EHDN Language Area Coordinators Portugal (J Ferreira, T Mestre), Spain (A Martínez Descals), France (A Durr, C Jauffret), The Netherlands (R Bos, R Roos, M-N Witjes-Ané), and England (R Fullam, O Handley, J Naji); HD Drug Works, Seattle, USA (L Goodman).

## Corresponding Author

Anthony L Vaccarino, avaccarino@ocbn.ca
